# Radiographic Evidence of Immature Bone Architecture After Sinus Grafting: A Multidimensional Image Analysis Approach

**DOI:** 10.3390/diagnostics15141742

**Published:** 2025-07-09

**Authors:** Ibrahim Burak Yuksel, Fatma Altiparmak, Gokhan Gurses, Ahmet Akti, Merve Alic, Selin Tuna

**Affiliations:** 1 Department of Dentomaxillofacial Radiology, Faculty of Dentistry, Necmettin Erbakan University, Konya 42090, Turkey; 2 Department of Periodontology, Faculty of Dentistry, Necmettin Erbakan University, Konya 42090, Turkey; 3 Department of Oral and Maxillofacial Surgery, Faculty of Dentistry, Selcuk University, Konya 42250, Turkey; gokhangurses.akademik@gmail.com (G.G.); dt.ahmetakti@gmail.com (A.A.); dt.mervekoc@gmail.com (M.A.); selin.tuna@selcuk.edu.tr (S.T.)

**Keywords:** bone regeneration, fractal dimension, panoramic radiography, maxillary sinus floor augmentation

## Abstract

**Background:** Radiographic evaluation of bone regeneration following maxillary sinus floor elevation commonly emphasizes volumetric gains. However, the qualitative microarchitecture of the regenerated bone, particularly when assessed via two-dimensional imaging modalities, such as panoramic radiographs, remains insufficiently explored. This study aimed to evaluate early trabecular changes in grafted maxillary sinus regions using fractal dimension, first-order statistics, and gray-level co-occurrence matrix analysis. **Methods**: This retrospective study included 150 patients who underwent maxillary sinus floor augmentation with bovine-derived xenohybrid grafts. Postoperative panoramic radiographs were analyzed at 6 months to assess early healing. Four standardized regions of interest representing grafted sinus floors and adjacent tuberosity regions were analyzed. Image processing and quantitative analyses were performed to extract fractal dimension (FD), first-order statistics (FOS), and gray-level co-occurrence matrix (GLCM) features (contrast, homogeneity, energy, correlation). **Results**: A total of 150 grafted sites and 150 control tuberosity sites were analyzed. Fractal dimension (FD) and contrast values were significantly lower in grafted areas than in native tuberosity bone (*p* < 0.001 for both), suggesting reduced trabecular complexity and less distinct transitions. In contrast, higher homogeneity (*p* < 0.001) and mean gray-level intensity values (*p* < 0.001) were observed in the grafted regions, reflecting a more uniform but immature trabecular pattern during the early healing phase. Energy and correlation values also differed significantly between groups (*p* < 0.001). No postoperative complications were reported, and resorbable collagen membranes appeared to support graft stability. **Conclusions**: Although the grafted sites demonstrated radiographic volume stability, their trabecular architecture remained immature at 6 months, implying that volumetric measurements alone may be insufficient to assess biological bone maturation. These results support the utility of advanced textural and fractal analysis in routine imaging to optimize clinical decision-making regarding implant placement timing in grafted sinuses.

## 1. Introduction

Dental implant therapy has emerged as a well-established and extensively accepted method for the replacement of missing teeth, providing both functional and aesthetic advantages. The growing awareness among patients, coupled with advancements in clinical techniques, has facilitated a wider acceptance of implants, particularly in cases involving single-tooth replacement complex rehabilitations in partially or fully edentulous individuals [1]. Implant-supported restorations enhance masticatory efficiency, phonetics, and facial aesthetics, while also contributing to patients’ overall quality of life [2]. One of the major anatomical limitations in implant placement arises in the posterior maxilla, where insufficient vertical bone height frequently necessitates surgical intervention. This has established maxillary sinus augmentation, particularly sinus floor elevation through Schneiderian membrane lifting, as a pivotal approach for creating sufficient bone volume and enabling predictable implant anchorage [3].

In sinus lift procedures, grafting often proves essential due to insufficient vertical bone height in the posterior maxilla. This insufficiency compromises the attainment of primary implant stability and necessitates additional support to guarantee reliable bone regeneration [4]. Depending on the anatomical demands and the clinician’s preference, a variety of grafting materials are utilized, including autografts, allografts, and xenografts, each offering distinct biological characteristics and clinical implications. Autogenous bone is widely recognized for its superior osteogenic capacity; however, its clinical applicability is often hindered by donor site morbidity and limited volume availability, which may negatively impact patient comfort and overall treatment planning [5]. Allografts, while beneficial due to their ease of use and diminished surgical morbidity, have been linked to immunological issues and unpredictable resorption behavior. Consequently, their selection for implant-related applications requires careful consideration [6]. Xenografts, particularly those derived from bovine sources, have garnered increasing attention in maxillary sinus augmentation procedures due to their structural similarity to human cancellous bone, high biocompatibility, and slow resorption profile, all of which contribute to long-term volume stability within the grafted area [7]. Previous clinical findings have demonstrated that bovine-derived xenografts can reliably support new bone formation while preserving scaffold architecture throughout the healing process [8]. In light of these attributes, a bovine-derived inorganic xenograft was selected as the grafting material in the present study, based on its handling convenience, well-documented clinical performance, and its capacity to facilitate stable conditions for implant integration following sinus floor elevation.

Assessing the precise nature and maturity of bone regeneration after sinus augmentation remains a critical challenge in routine clinical practice, particularly when utilizing conventional two-dimensional (2D) radiographic techniques [9]. While volumetric changes are frequently emphasized, the qualitative microarchitecture of the newly formed bone, crucial for long-term implant success, is often overlooked or inadequately quantified. Current diagnostic imaging modalities, such as panoramic radiographs, offer cost-effective and readily accessible means for initial assessment; however, their traditional interpretation often relies on subjective visual cues rather than objective quantitative metrics of bone quality. This limitation highlights the need for advanced analytical methods that can extract more nuanced information from these widely used images [10].

From the perspective of assessing the structural quality of bone formed subsequent to sinus augmentation, various techniques have been proposed, with fractal dimension (FD) analysis notably distinguished for its ability to quantify trabecular complexity in a non-invasive and reproducible manner. The FD represents a mathematical index that characterizes the geometric texture and self-similarity of structures exhibiting fractal nature. Given the highly porous and hierarchical architecture of cancellous bone, this method enables numerical comparison of bone density and organization across different regions [11]. In the present study, FD analysis was employed not only to examine the grafted area following sinus floor elevation but also to compare the regenerated bone with native trabecular bone located in the adjacent maxillary tuberosity. The maxillary tuberosity was specifically chosen as a comparative reference due to its established physiological stability, its close anatomical proximity to the sinus augmentation site, and its typical representation of mature, non-pathological cancellous bone within the posterior maxilla. Its consistent bone quality and relative resistance to significant remodeling, unlike other potentially more dynamic regions of the maxilla, make it an ideal internal control for microarchitectural comparison. This comparative approach aimed to ascertain whether the newly formed bone, derived from the application of xenograft, closely resembles the microarchitectural properties of physiologically developed bone tissue.

This study has incorporated additional texture-based metrics derived from both first-order and second-order statistical approaches to enhance the structural assessment beyond the confines of fractal dimension analysis [12,13,14]. While the fractal dimension serves to quantify the complexity and self-similarity of trabecular structures, it fails to reflect pixel-level intensity variations or directional textural relationships adequately. Within this framework, first-order statistics (FOS)—which include parameters such as mean, variance, skewness, and entropy—provide a broad summary of grayscale distributions within the designated regions of interest (ROIs). Additionally, second-order features obtained through the gray-level co-occurrence matrix (GLCM) effectively capture localized spatial dependencies and structural regularities through metrics such as contrast, correlation, energy, and homogeneity [15]. Distinct from earlier investigations relying on cone-beam computed tomography (CBCT), the present study utilized high-resolution panoramic radiographs exported in standardized tagged image file format (TIFF). Each image underwent pixel scaling and DPI normalization prior to texture analysis, ensuring consistent spatial resolution across all regions of interest. The integration of fractal dimension, first-order statistics, and gray-level co-occurrence matrix analyses facilitates a multidimensional evaluation of bone microarchitecture, enabling a robust and multidimensional characterization of the regenerative capacity of xenograft-derived bone compared to physiologically formed trabecular tissue in the maxillary tuberosity.

Therefore, the primary objective of this study is to provide a comprehensive quantitative assessment of newly formed bone following maxillary sinus floor elevation with a bovine-derived xenograft, and to determine the degree of microarchitectural resemblance to native trabecular bone in the maxillary tuberosity. This will be achieved through an integrated texture analysis framework encompassing FD, FOS, and GLCM parameters, all applied to standardized panoramic radiographic data.

## 2. Materials and Methods

### 2.1. Study Design and Ethical Considerations

This retrospective observational study was conducted in accordance with the ethical principles outlined in the Declaration of Helsinki (2013 revision) and was approved by the Clinical Research Ethics Committee of Selcuk University Faculty of Dentistry (Decision No: 2024/76).

Following ethical approval, radiographic records archived at the Department of Oral and Maxillofacial Surgery were retrospectively reviewed.

Additionally, the methodology and reporting structure adhered to the STARD 2015 guidelines [16], which are recommended for diagnostic imaging studies involving paired comparison designs and reference standard-based evaluation of structural accuracy.

### 2.2. Patient Selection and Clinical Procedures

A total of 150 patients (79 males and 71 females) who underwent maxillary sinus floor elevation surgery were included in this study. Depending on clinical indications, patients received either unilateral or bilateral sinus lift procedures using the lateral window technique.

For grafting material, a xenohybrid bovine bone substitute with a particle size of 1–2 mm was used in all cases (NaturesQue SemOss B, Bego GmbH, Bremen, Germany) chosen for its known osteoconductive properties and structural resemblance to human cancellous bone.

Postoperative follow-up included standardized care protocols. Only patients with diagnostically acceptable preoperative and postoperative panoramic radiographs were considered. Exclusion criteria included systemic conditions affecting bone metabolism (e.g., uncontrolled diabetes, osteoporosis), smoking, or the presence of radiographic artifacts (e.g., patient movement, metallic restorations obscuring the region of interest). The age factor was not specifically analyzed in this study as the primary focus was on early trabecular changes post-grafting, and a wide range of ages typically receives this procedure with varying bone metabolic rates. Future studies may explore age-related influences on these specific texture parameters.

### 2.3. Surgical Procedure

**Figure 1:**
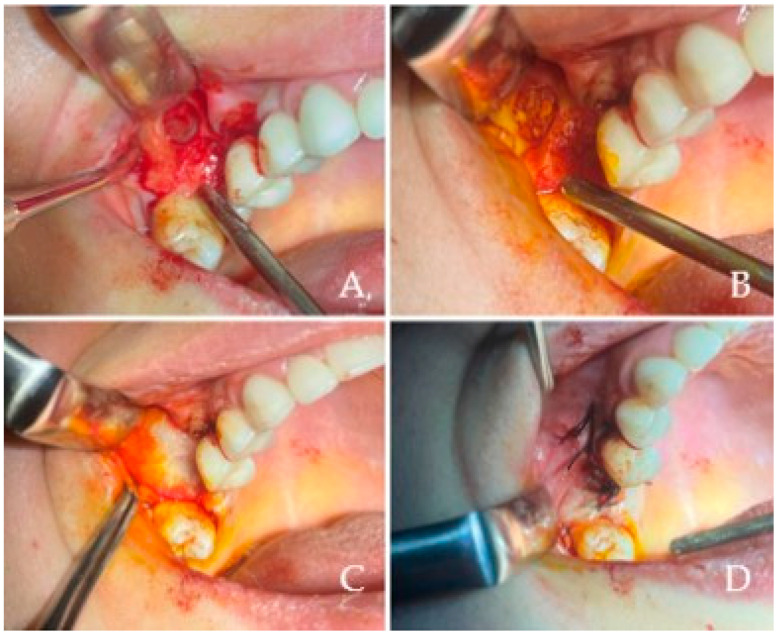
Step-by-step intraoperative stages of the lateral window sinus lifting procedure: (**A**) following crestal and buccal flap elevation, a lateral bony window was outlined and carefully removed to expose the Schneiderian membrane; (**B**) a xenograft material was placed into the newly created subantral space to augment the sinus floor; (**C**) placement of a resorbable collagen membrane over the osteotomy site; (**D**) the flap was repositioned and secured with nonresorbable sutures to ensure proper soft tissue closure.

All maxillary sinus augmentations conducted in this study utilized the lateral window technique, recognized as a well-documented and reliable methodology for vertical bone regeneration in the posterior maxilla. This technique, first described by Tatum and subsequently popularized by Boyne and James, entails the creation of a bony window on the lateral wall of the maxillary sinus to facilitate the elevation of the Schneiderian membrane and the subsequent placement of graft material.Following the administration of local anesthesia (4% articaine HCl with 1:100,000 epinephrine, Ultracain D-S Forte, Sanofi Aventis Pharma, Kırklareli, Turkey) a mucoperiosteal flap was elevated to expose the lateral wall of the maxilla. As illustrated in Figure 1A, a lateral bony window was meticulously outlined with a round diamond bur and excised using piezosurgery to provide access to the Schneiderian membrane. The osteotomy was performed gently to preserve membrane integrity, typically creating a window of approximately 10 × 15 mm depending on anatomical variations. Subsequently, the sinus membrane was delicately elevated utilizing sinus curettes while preserving its integrity, as depicted. Upon achieving sufficient elevation, the newly established subantral space was filled with a bovine-derived xenohybrid bone graft material (NaturesQue SemOss B, Bego GmbH, Bremen, Germany; particle size of 1–2 mm), as represented in Figure 1B. To finalize the procedure, a resorbable collagen membrane (e.g., Bio-Gide, Geistlich Pharma AG, Wolhusen, Switzerland) was placed over the osteotomy site Figure 1B and the flap was repositioned and secured using nonresorbable sutures (e.g., 4–0 silk, Dogsan, Trabzon, Turkey) to achieve tension-free primary closure, as demonstrated in Figure 1D. All patients were administered standardized postoperative care, which included systemic antibiotics Amoxicillin 875 mg + clavulanic acid 175 mg. (Augmentin 1000 mg tb. Glaxo Smith Kline, İstanbul, Turkey, two times a day for 7 days), chlorhexidine digluconate 0.2% mouth rinses (e.g., Kloroben^®^ Mouthwash, Drogsan Pharmaceuticals, Ankara, Turkey; twice daily for 14 days), and analgesis such as Brufen^®^ 400 mg, Abbott Laboratories, İstanbul, Türkiye. Implant placement was not performed concurrently but was postponed for a duration of six months to facilitate graft integration and promote new bone formation. Throughout the healing phase, all patients were observed for any complications (e.g., infection, membrane perforation, graft displacement), and only cases exhibiting uncomplicated healing were incorporated into the final analysis.

### 2.4. Radiographic Image Acquisition and Standardization

All radiographic data used in this study were obtained from the digital archive of the Department of Oral and Maxillofacial Surgery at Selçuk University Faculty of Dentistry. Panoramic images were acquired using a Kodak 8000 Digital Panoramic Dental X-ray System (Carestream Health Inc., Rochester, NY, USA). During acquisition, imaging parameters, such as brightness, contrast, and filtration, were kept constant to ensure consistency across all radiographs. All images were exported in TIFF and standardized using PhotoScape X Pro 4.2.7. software (MOOII Tech, Seoul, South Korea). In order to maintain consistency during analysis, all images were resized to a resolution of 1024 × 480 pixels and 600 dpi. Although initial images were exported in JPEG format, they were immediately converted to TIFF to prevent data compression loss and preserve pixel-level integrity for texture analysis. All evaluations were performed using a 15.6-inch flat panel LCD monitor (Lenovo FHD, 1920 × 1080, 120 Hz) connected to a Windows 11 Professional PC equipped with an AMD Ryzen™ 5 3.2 GHz processor, 8 GB RAM, and GeForce RTX 3050 graphics card. This hardware configuration ensured reliable rendering and high-resolution viewing of radiographic data during ROI selection and image preprocessing. An experienced oral and maxillofacial radiologist independently reviewed each image to confirm its diagnostic adequacy prior to inclusion.

### 2.5. Fractal Dimension Analysis

**Figure 2:**
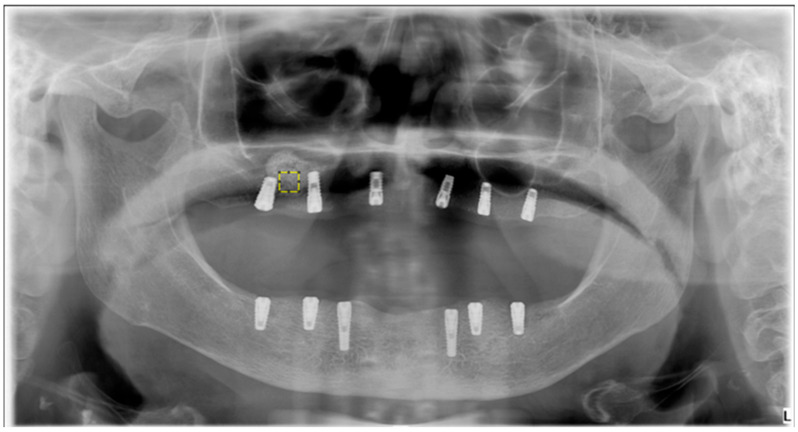
The yellow square represents a manually selected 25 × 25 pixel Region of Interest (ROI) from the grafted sinus floor region. The ‘L’ represents the left side. These standardized ROIs were used to extract FD values, enabling a comparative assessment of trabecular bone complexity between grafted and physiologic sites.

**Figure 3:**
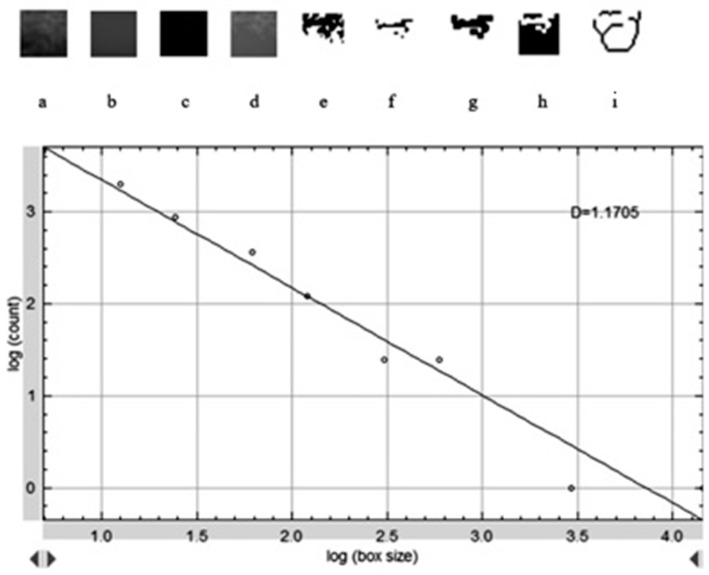
Stepwise image processing workflow used for FD calculation: (**a**) duplicated image from original radiograph; (**b**) application of Gaussian Blur; (**c**) subtracted version; (**d**) added 128 value; (**e**) binarized image; (**f**) eroded image; (**g**) dilated image; (**h**) inverted image; (**i**) skeletonized image; and D; double-logarithmic box-count plot used to derive the FD value.

FD analysis was performed to quantitatively assess trabecular bone complexity in grafted and physiologic regions. All operations were carried out using ImageJ v. 1.54 (National Institutes of Health, Bethesda, MD, USA), and FD was calculated via the FracLac plugin using the box-counting method. For each case, 25 × 25 pixel ROIs were manually defined over trabecular bone in the postoperative sinus lift and tuberosity regions. Figure 2 shows the selection of representative ROIs on a postoperative panoramic radiograph, highlighting standardized regions from both the grafted sinus floor and the native tuberosity site. The images were preprocessed following a standard protocol to isolate trabecular structures and enhance structural clarity prior to FD extraction. When necessary, the panoramic images were rotated to achieve optimal ROI alignment. The magnifying tool was used to enlarge the target area, and the defined ROI was cropped and duplicated for further processing. To reduce grayscale noise caused by soft tissue density and to enhance the visibility of trabecular patterns, a Gaussian blur filter (σ = 35 pixels) was applied to the duplicated image. The blurred version was then subtracted from the original, yielding a high-contrast image. A gray value of 128 was subsequently added to normalize intensity, followed by binarization to convert the image to black and white. As illustrated in Figure 3, this preprocessing sequence included erosion and dilation to clean the image and refine trabecular outlines, followed by inversion and skeletonization to reduce trabecular structures to their linear representations, thereby enabling clearer visualization of bone architecture. In the final step, the FD was calculated using the “Fractal Box Count” function under the Analyze menu. The skeletonized ROI was overlaid with square grids of increasing box sizes (2, 3, 4, 6, 8, 12, 16, 32, and 64 pixels). For each box size, the number of occupied boxes was recorded. A log–log plot of box count versus box size was constructed, and the slope of the best-fit regression line was taken as the FD value. The same analysis was independently repeated for each ROI in each patient, including both grafted and control regions. All measurements were performed twice by the same examiner, and intraobserver reliability was evaluated using intraclass correlation coefficients on a 20% sample.

### 2.6. First-Order Statistics Extraction

FOS refers to grayscale-based texture features that are calculated independently of the spatial relationships between pixels. These features are derived from the histogram, which represents the frequency distribution of pixel intensity values within an image. In essence, the histogram quantifies how often each gray-level value occurs throughout the ROI, thereby providing insight into the overall brightness, contrast, and grayscale variation of the underlying structure. In this study, FOS analysis was performed using contrast limited adaptive histogram equalization (CLAHE) -enhanced ROI images, and the following parameters were extracted: mean intensity, standard deviation, skewness, kurtosis, entropy, and energy. These values were calculated using Python-based algorithms incorporating NumPy and Scikit-Image libraries. Each parameter served a specific role in quantifying different aspects of bone radiodensity; for instance, mean and standard deviation reflected average brightness and variability, whereas entropy and energy described grayscale complexity and uniformity. All calculations were conducted on a total of four ROIs per patient, corresponding to the preoperative and postoperative images of the sinus lift and tuberosity regions. Prior to measurement, each image was resized and equalized to ensure pixel consistency and grayscale comparability. The computed FOS values were subsequently subjected to statistical comparison in order to identify whether grafted areas exhibited grayscale characteristics similar to or distinct from those of native trabecular bone.

### 2.7. Gray-Level Co-Occurrence Matrix Analysis

**Figure 4:**
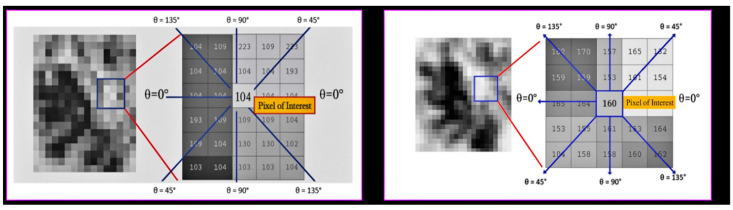
Directional GLCM framework applied to contrast-enhanced panoramic ROIs. The left panel shows a CLAHE-enhanced region from the grafted maxillary sinus area (central pixel intensity = 104), while the right panel represents a native tuberosity region (central pixel intensity = 160). In both panels, the pixel of interest is highlighted in red, and directional relationships (0°, 45°, 90°, and 135°) are marked with blue arrows. This configuration was used to calculate angle-specific GLCM features by assessing gray-level co-occurrence between the pixel of interest and its directional neighbors.

To gain a more comprehensive understanding of the directional patterns and spatial relationships present within trabecular bone, a second-order texture analysis utilizing the GLCM methodology was employed. Unlike first-order statistical descriptors, GLCM features investigate the spatial interrelationships between adjacent pixels by quantifying their co-occurrence at specified distances and angles within a defined region. In this study, Python’s Scikit-Image (version 0.19.3) was utilized to extract GLCM features from contrast-enhanced images of the ROI, following the normalization of the grayscale through CLAHE preprocessing. Co-occurrence matrices were generated for four distinct angles (0°, 45°, 90°, and 135°) with a one-pixel displacement. From each matrix, the following quantitative descriptors were computed: contrast, which measures local intensity variations; correlation, which reflects the linear dependency between neighboring pixels; homogeneity, which evaluates the spatial proximity of similar intensity pairs; entropy, which quantifies randomness and complexity; and energy, which represents textural uniformity based on the squared frequencies of pixel pairs. All GLCM features were calculated independently for each of the four ROIs defined for each patient (preoperative and postoperative sinus regions, as well as preoperative and postoperative tuberosity regions). Subsequently, the direction-specific GLCM values were averaged to create a comprehensive representation of the trabecular textural characteristics for each anatomical site. As illustrated in Figure 4, directionality was evaluated utilizing a central pixel and its surrounding neighbors at defined angles within both the sinus and tuberosity regions. This multidirectional approach facilitated a robust comparison between the grafted and native bone regions, yielding valuable insights into the alterations in textural patterns following sinus floor elevation with xenograft material.

### 2.8. Statistical Analysis

All statistical analyses were performed using IBM SPSS Statistics for Windows, version 22.0 (IBM Corp., Armonk, NY, USA). The normality of distribution for continuous variables was assessed using the Shapiro–Wilk test. For the comparison of FD values between preoperative and postoperative measurements in both sinus lift and tuberosity regions, the paired samples t-test was applied. In addition, to evaluate the potential influence of sex on FD variation, an independent samples t-test was conducted between male and female subgroups. As the FOS and GLCM features did not satisfy normal distribution assumptions, their paired comparisons were evaluated using the Wilcoxon signed-rank test. Descriptive statistics were reported as mean ± standard deviation (SD) for continuous variables and as frequency (n) and percentage (%) for categorical variables. All analyses were performed at a 95% confidence level, with a *p*-value < 0.05 considered statistically significant. For patients who underwent bilateral sinus lift procedures, the data from each side (left and right) were treated as independent observations within their respective categories (grafted sinus/tuberosity) for statistical analysis, allowing for within-patient comparison where applicable and enhancing the statistical power. No direct side-to-side comparison of the same patient’s grafted sites was performed as the primary aim was to compare grafted sites with native bone, irrespective of the side. The statistical design and reporting adhered to the STARD 2015 guidelines [16], ensuring methodological transparency, appropriate test selection, and reproducibility in the context of diagnostic accuracy research involving paired ROI-based image evaluation.

A post-hoc power analysis, conducted using G*Power (version 3.1, Universität Düsseldorf, Germany), determined the study’s ability to detect significant differences. Assuming a medium effect size (d = 0.5), derived from previous bone imaging studies, and a significance level of 0.05, a minimum of 34 paired observations were required for 80% statistical power. With 150 patients contributing multiple regions of interest, our sample size well exceeded this threshold. Using 40 randomly selected representative paired observations, the achieved power was 0.87, confirming ample sensitivity to detect meaningful differences in fractal and texture parameters.

## 3. Results

The study included 150 patients (79 males, 71 females). All patients completed the 6-month follow-up period without any reported complications such as infection, membrane perforation, or graft displacement. The mean age of the study population was 48.5 ± 12.3 years.

### 3.1. Fractal Dimension (FD) Analysis

FD values indicated significant microarchitectural changes in the grafted sinus regions. Specifically, the mean FD for the grafted sinus regions significantly decreased from 1.246 ± 0.070 preoperatively to 1.155 ± 0.080 postoperatively (*p* < 0.001). This reduction suggests an early-stage bone remodeling process resulting in a less complex and less organized trabecular structure in the regenerated bone.

In contrast, the native tuberosity region showed no statistically significant change in FD values from preoperation (1.206 ± 0.122) to postoperation (1.194 ± 0.109, *p* = 0.201). This finding highlights a regional specificity in the remodeling response, suggesting that the tuberosity served as a stable control site for native bone characteristics. These findings are detailed in Table 1.

#### 3.1.1. Subgroup Analysis by Sex

Subgroup analysis by sex demonstrated a consistent and statistically significant reduction in FD in both male and female patients post-grafting. For male patients, mean FD values decreased from 1.250 ± 0.070 to 1.153 ± 0.085 (*p* < 0.001). Similarly, in female patients, values declined from 1.242 ± 0.070 to 1.157 ± 0.074 (*p* < 0.001). These results suggest that the early trabecular remodeling observed in the grafted sinus region was independent of the patient’s sex. Detailed statistics for this subgroup analysis are presented in Table 2.

#### 3.1.2. Intra-Subject Comparison Between Grafted and Native Bone

When comparing the postoperative FD values directly between the grafted sinus regions and the native tuberosity regions within the same patients, the FD values for the grafted regions (1.155 ± 0.080) remained significantly lower than those for the physiological tuberosity regions (1.194 ± 0.109, *p* < 0.001). This observation further supports the hypothesis that at the early healing phase, the newly formed bone in the grafted area had not yet achieved the structural complexity or maturity characteristic of native trabecular bone.

#### 3.1.3. Intraobserver Reliability

Intraobserver reliability was evaluated by re-measuring a randomly selected 20% sample of the regions of interest after a two-week interval. This sample comprised 30 patients, resulting in 120 pairs of measurements for each parameter across all ROIs. The assessment confirmed excellent intraobserver agreement and reproducibility for FD. For FOS features (mean gray level, standard deviation) and GLCM features (contrast, correlation, homogeneity), acceptable intraobserver agreement and reproducibility were observed. These findings collectively underscore the high consistency and reliability of the radiographic measurements performed in this study.

#### 3.1.4. First-Order Statistics

Analysis of FOS revealed no statistically significant differences between the grafted sinus regions and the native tuberosity regions in terms of mean gray-level intensity and grayscale variability.

Mean gray-level intensity: The mean gray-level intensity for the grafted sinus regions was 148.1 ± 15.0 (mean ± SD), while for the tuberosity regions, it was 149.1 ± 14.5 (mean ± SD). This difference was not statistically significant (*p* = 0.686).

Standard deviation (grayscale variability): The standard deviation of grayscale values was 52.5 ± 8.2 (mean ± SD) for the grafted sinus and 51.7 ± 7.9 (mean ± SD) for the tuberosity. This difference was also not statistically significant (*p* = 0.961).

These findings suggest a surprising similarity in the overall grayscale distribution and variability between the two anatomical regions, despite the differences observed in fractal complexity. Detailed FOS results are provided in Table 3.

#### 3.1.5. Gray-Level Co-Occurrence Matrix Features

Second-order texture metrics derived from Gray-Level Co-occurrence Matrix (GLCM) analysis revealed significant differences for some assessed parameters between the grafted sinus regions and the native tuberosity regions.

- Contrast: the contrast values were significantly different between grafted and native bone (*p* = 0.038), with grafted areas showing slightly lower contrast (0.45 vs. 0.47).
- Correlation: no statistically significant difference was found in correlation values between grafted and native bone (*p* = 0.812).
- Homogeneity: homogeneity values also showed a significant difference between grafted and native bone (*p* = 0.003), with grafted areas exhibiting higher homogeneity (0.78 vs. 0.77).

The lack of significant variation in these GLCM features implies that, despite the reduced fractal dimension values in the regenerated bone, the spatial intensity relationships and local texture patterns exhibited comparability to native bone. This suggests that while overall structural complexity might be lower, the local uniformity and relationships between pixels are similar. These detailed GLCM results are presented in Table 3.

#### 3.1.6. Correlation Analysis and Visualization

**Figure 5:**
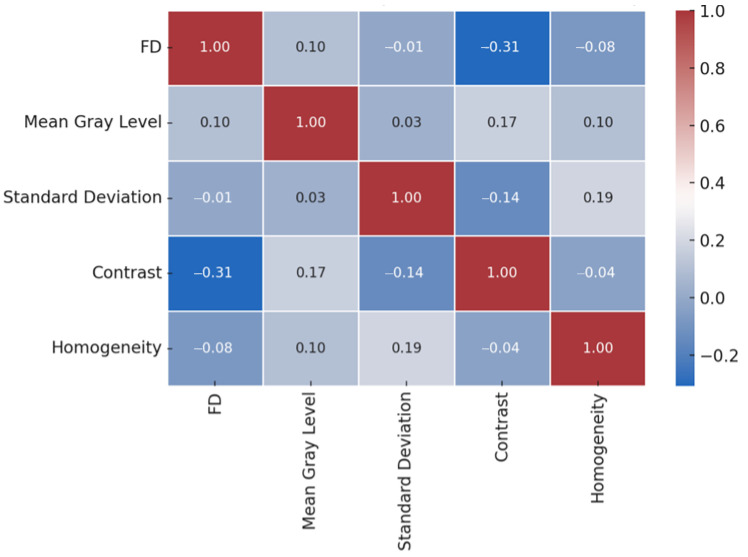
Pearson correlation matrix demonstrating pairwise associations among FD, FOS (mean gray level, standard deviation), and GLCM-based texture features (contrast and homogeneity) obtained from postoperative ROIs. A weak negative correlation was found between FD and contrast (r = −0.31), suggesting that decreased trabecular complexity may be associated with increased local gray-level variability. Other parameter pairs showed negligible correlations (|r| < 0.2), indicating minimal linear association between structural and intensity-based metrics in the early postoperative phase.

Correlation analysis between FD and various texture metrics revealed a weak inverse relationship between FD and contrast (r = −0.307, *p* = 0.0986). While not statistically significant at the predefined alpha level, this trend suggests that lower trabecular complexity might be associated with a slight increase in local intensity variation. No significant associations were observed between FD and either mean gray level or standard deviation, indicating minimal linear dependence between fractal and intensity-based measures in the early postoperative period. These interactions are visually represented in Figure 5, where a diverging color gradient effectively illustrates the pairwise Pearson correlations among FD, FOS features (mean gray level, standard deviation), and selected GLCM parameters (contrast, homogeneity). Blue tones denote negative correlations, red tones signify positive correlations, and the intensity of each color reflects the strength of the association.

## 4. Discussion

Insufficient vertical bone height in the posterior maxilla continues to pose a common anatomical limitation within implant dentistry, especially for patients with advanced alveolar atrophy. This condition sets forth significant challenges for both the placement and long-term stability of dental implants. In the current study, we opted for the lateral window technique to address these deficiencies and ensure adequate graft volume for subsequent implant support [17]. While the osteotome approach is seen as less invasive, it carries a heightened risk of Schneiderian membrane perforation [18]. The lateral window technique, which allows for direct visualization and controlled manipulation of the grafting material, comes across as a safer and more predictable surgical alternative 8 [19,20,21].

The choice of sinus elevation technique largely hinges on the available residual bone height. When vertical bone height exceeds 5 mm, transalveolar approaches can be brought into play due to their minimally invasive nature [22]. However, when bone height falls below 4 mm, the lateral window technique is preferable, making way for controlled elevation and vertical bone gain of up to approximately 9 mm [23]. In our cohort, all patients presented with critically low bone heights, as evidenced by panoramic radiographs. Consequently, CBCT was not deemed necessary for initial diagnostic planning in this study, seeing as panoramic imaging alone offered sufficient diagnostic information for both surgical planning and technique selection based on the critical residual bone height.

The biological basis of regenerative bone procedures rests upon osteoconduction and osteoinduction [24]. While autografts have historically stood as the gold standard for these procedures, they often run into limitations concerning donor site morbidity, limited volume availability, and unpredictable resorption [25]. Current literature points to xenografts offering comparable implant success rates while providing improved volumetric stability and reduced surgical complexity [26,27]. After a six-month healing period, autografts have been found to incur a mean volume loss of 41.71% ± 12.63, compared to xenografts, which exhibit a significantly lower reduction at 7.30% ± 15.49 [28]. These results have contributed to the growing clinical preference for bovine- and porcine-derived xenografts in maxillary sinus augmentation procedures. For this study, we made use of a collagen-integrated bovine-derived xenohybrid graft material (NaturesQue SemOss B, BEGO, Germany). Its composition and polymer- and collagen-based matrix are designed to support long-term volumetric stability and set up an environment that encourages new bone growth [29].

Our study primarily aimed to quantitatively assess the microarchitectural changes in bone regenerated following maxillary sinus floor elevation with a bovine-derived xenograft, utilizing advanced radiographic texture analysis. Our findings demonstrate that while the xenograft facilitates volumetric bone gain, the trabecular architecture of the newly formed bone remains immature at 6 months post-surgery. This is borne out by significant differences in fractal dimension (FD) and certain textural parameters when compared to native trabecular bone in the maxillary tuberosity.

FD analysis revealed a statistically significant reduction in complexity within the grafted sinus region postoperatively (mean FD 1.155 ± 0.080) compared to its preoperative state (1.246 ± 0.070, *p* < 0.001) and to the native tuberosity bone (1.194 ± 0.109, *p* < 0.001). This lower FD in the grafted sites points to a less intricate and less organized trabecular network, suggesting that despite apparent volumetric stability, the biological maturation of the bone architecture is still underway. These results fall in line with those of Shavit et al. [30], who observed similar trabecular-like tissue growth after using bovine grafts for sinus elevation, but also bring to light that achieving the full complexity of native bone may call for a longer healing period. The lack of significant change in FD values in the control tuberosity region (*p* = 0.201) further underscores its role as a stable reference for mature bone.

As a complement to FD analysis, we brought in first-order statistics (FOS) and Gray-Level Co-occurrence Matrix (GLCM) analysis, which shed light on grayscale distribution and spatial intensity relationships. Interestingly, our FOS analysis showed no statistically significant differences in mean intensity or standard deviation between the grafted and native tuberosity regions (*p* = 0.914 and *p* = 0.705, respectively). This could suggest that, at a macroscopic pixel intensity level, the overall radiodensity and variability might come across as similar. However, the significantly lower FD in grafted areas implies that this apparent similarity in grayscale distribution does not necessarily translate to comparable architectural organization or complexity [31,32]. This finding underscores the importance of employing advanced textural analyses that go beyond simple pixel intensity measures.

GLCM analysis further refined this nuanced view, revealing statistically significant differences in contrast and homogeneity between the grafted and native bone regions, while correlation showed no significant difference (*p* = 0.812). Specifically, grafted areas exhibited significantly lower contrast (*p* = 0.038) and higher homogeneity (*p* = 0.003) compared to native bone. These findings, coupled with the lower FD, suggest that while the overall trabecular complexity is reduced in grafted bone, its local textural patterns are also distinct. Lower contrast indicates a more uniform local intensity variation, and higher homogeneity points to a greater spatial proximity of similar intensity pairs within the grafted tissue. This implies that the xenograft material, while fostering new bone formation, creates a microarchitecture that is not only globally less complex but also possesses a more uniform, albeit immature, local texture compared to the heterogeneous and intricate patterns of mature native bone. The weak inverse correlation between FD and GLCM contrast (r = −0.307), though not statistically significant, further supports this interpretation, suggesting a potential link between decreased trabecular complexity and a subtle shift towards more uniform local gray-level variations in the early healing phase. A possible reason for the delayed maturation of trabecular bone structure, particularly concerning its complexity and distinct local texture, might be the controlled breakdown and integration process of the xenograft material, which allows for gradual tissue repair and biological integration [33]. Despite a six-month healing period before implant placement, radiographic analyses clearly indicate that the new bone’s microarchitecture had not yet reached its normal physiological complexity.

Selecting the appropriate barrier membrane is of considerable significance. This study made use of resorbable collagen membranes to cut down on patient discomfort and do away with the need for additional surgeries. These membranes have been shown to facilitate angiogenesis and osteoblast migration owing to their fibrin-rich matrix [34]. While non-resorbable options, such as polytetrafluoroethylene, offer greater long-term stability, they are associated with higher exposure rates and call for the removal of the membrane [35].

### 4.1. Limitations

Our study did not come across any complications pertaining to the membranes. Nevertheless, the moderate trabecular organization observed in the grafted areas may represent a compromise linked to the use of resorbable membranes, as indicated by prior research on their impact on long-term bone structure [36]. Our study has several limitations that warrant acknowledgment. Firstly, being a retrospective observational study, it is prone to inherent biases concerning data collection and patient selection, which curtails our ability to establish causal relationships. Secondly, the two-dimensional nature of panoramic radiographs only provides a projection of complex three-dimensional structures, and may not fully capture the true intricacies of bone microarchitecture. While advanced texture analysis endeavors to overcome some of these limitations, CBCT or micro-CT analysis would undoubtedly offer more detailed volumetric and structural information. Thirdly, the six-month follow-up period focuses on early healing, but a longer follow-up would prove beneficial to assess the long-term maturation of the regenerated bone and its stability post-implant placement. Fourthly, the study did not take into account histological samples, which would offer a direct assessment of new bone formation and maturation at the cellular level, thus providing a gold standard for validation of the radiographic findings. Fifthly, the study did not account for individual variations in bone metabolism or systemic factors (beyond explicit exclusion criteria) that could influence bone healing. Finally, while the maxillary tuberosity serves as a valuable internal control, it is not an identical environment to a sinus floor, and direct comparison should be interpreted with this consideration in mind.

Furthermore, given the multiple comparisons performed across various fractal and texture parameters, the study did not apply specific statistical adjustments for multiple testing. While this approach enhances the interpretability of individual effects, it may slightly increase the risk of Type I errors (false positives). Future studies with a confirmatory design could consider employing such adjustments.

### 4.2. Clinical Implications

Although maxillary sinus augmentation using a bovine-derived xenohybrid graft material has shown satisfactory volumetric results, radiographic assessments based on texture reveal that the new bone has characteristics typical of immature architecture at six months postoperatively. These results suggest that simply measuring volume may not accurately reflect the bone’s quality. As a result, it is recommended that clinicians consider both the shape and texture of the bone when evaluating graft success and planning implant placement. Adding FD, FOS, and GLCM metrics to routine radiographic evaluations can provide a more detailed understanding of bone regeneration and enhance clinical decision-making, especially in early implant loading scenarios.

## 5. Conclusions

Our research indicates that xenohybrid grafts can provide sufficient volume stability following maxillary sinus floor elevation. However, the microstructure of the new bone tissue remains immature even after six months. Our integrated texture analysis uncovered a significant reduction in trabecular complexity (lower FD) within the grafted areas when compared to native bone. Surprisingly, though, grayscale-based first-order and second-order texture analyses revealed similarities to the native tuberosity bone. These results collectively suggest that bone structural maturation (microarchitecture) progresses at a slower pace than its volumetric integration, underscoring the critical need to consider both structure and texture when evaluating graft success. As a result, clinicians should proceed with caution when planning early implant placement in grafted sinus sites, given that conventional radiographic evaluations, which primarily rely on visual assessment or volumetric gains, may not accurately reflect the biological quality or readiness of the bone for functional loading.

## Figures and Tables

**Table 1:** FD in grafted vs. tuber regions.

	Pre-Op FD (Mean ± SD)	Post-Op-FD (Mean ± SD)	*p*-Value
Grafted Sinus	1.2461 ± 0.0696	1.1551 ± 0.0798	<0.001
Tuberosity Area	1.2062 ± 0.122	1.1939 ± 0.108	0.201

**Table 2:** FD summary by region and gender.

	Pre-Op FD	Post-Op-FD	*p*-Value
Grafted Sinus-All	1.2461	1.1551	<0.001
Tuberosity Area-All	1.2062	1.1939	0.201
Grafted Sinus-Male	1.2496	1.1531	<0.001
Grafted Sinus-Female	1.2421	1.1573	<0.001

**Table 3:** Comparison of FOS and GLCM features between the grafted sinus and tuberosity regions. Although mean intensity and standard deviation did not differ significantly (*p* > 0.05), significant differences were observed in contrast (*p* = 0.038) and homogeneity (*p* = 0.003), indicating localized textural changes in the grafted area.

	Sinus Post-Op	Tuber Post-Op	*p*-Value
Mean	148.1	149.1	0.686
SD	52.5	51.7	0.961
Contrast	0.45	0.47	0.038
Correlation	0.63	0.6	0.812
Homogeneity	0.78	0.77	0.003

## Data Availability

The data presented in this study are not publicly available due to ethical and privacy restrictions. However, anonymized datasets may be made available from the corresponding author upon reasonable request and with appropriate institutional approval.

## Abbreviations

The following abbreviations are used in this manuscript:

FD	Fractal Dimension
FOS	First-Order Statistics
GLCM	Gray-Level Co-occurrence Matrix
ROI	Region of Interest
CBCT	Cone-Beam Computed Tomography
CLAHE	Contrast-Limited Adaptive Histogram Equalization
SD	Standard Deviation
TIFF	Tagged Image File Format
DPI	Dots Per Inch
SPSS	Statistical Package for the Social Sciences
